# -Omic Approaches and Treatment Response in Rheumatoid Arthritis

**DOI:** 10.3390/pharmaceutics14081648

**Published:** 2022-08-08

**Authors:** Adela Madrid-Paredes, Javier Martín, Ana Márquez

**Affiliations:** 1 Department of Clinical Pharmacy, San Cecilio University Hospital, Instituto de Investigación Biosanitaria de Granada (ibs.Granada), 18016 Granada, Spain; 2 Institute of Parasitology and Biomedicine López-Neyra (IPBLN), Spanish National Research Council (CSIC), 18017 Granada, Spain

**Keywords:** epigenomics, DNA methylation, microRNAs, genomics, transcriptomics, proteomics, treatment response, rheumatoid arthritis

## Abstract

Rheumatoid arthritis (RA) is an inflammatory disorder characterized by an aberrant activation of innate and adaptive immune cells. There are different drugs used for the management of RA, including disease-modifying antirheumatic drugs (DMARDs). However, a significant percentage of RA patients do not initially respond to DMARDs. This interindividual variation in drug response is caused by a combination of environmental, genetic and epigenetic factors. In this sense, recent -omic studies have evidenced different molecular signatures involved in this lack of response. The aim of this review is to provide an updated overview of the potential role of -omic approaches, specifically genomics, epigenomics, transcriptomics, and proteomics, to identify molecular biomarkers to predict the clinical efficacy of therapies currently used in this disorder. Despite the great effort carried out in recent years, to date, there are still no validated biomarkers of response to the drugs currently used in RA. -Omic studies have evidenced significant differences in the molecular profiles associated with treatment response for the different drugs used in RA as well as for different cell types. Therefore, global and cell type-specific -omic studies analyzing response to the complete therapeutical arsenal used in RA, including less studied therapies, such as sarilumab and JAK inhibitors, are greatly needed.

## 1. Introduction

Rheumatoid arthritis (RA), which affects approximately 0.5–1% of the world population, is a chronic and progressive inflammatory disorder characterized by the appearance of synovitis and severe joint destruction that causes pain and disability [1,2]. The appearance of RA is related to the subsequent activation and proliferation of both innate and adaptive immune cells, including neutrophils, T and B lymphocytes, and monocytes, as well as fibroblasts of the synovial membrane, thus leading to persistent inflammation and damage of joints and bones [3]. This aberrant cell activation is mediated by genetic and environmental factors as well as by epigenetic factors, which represent the link between genetic and environment.

**Figure 1:**
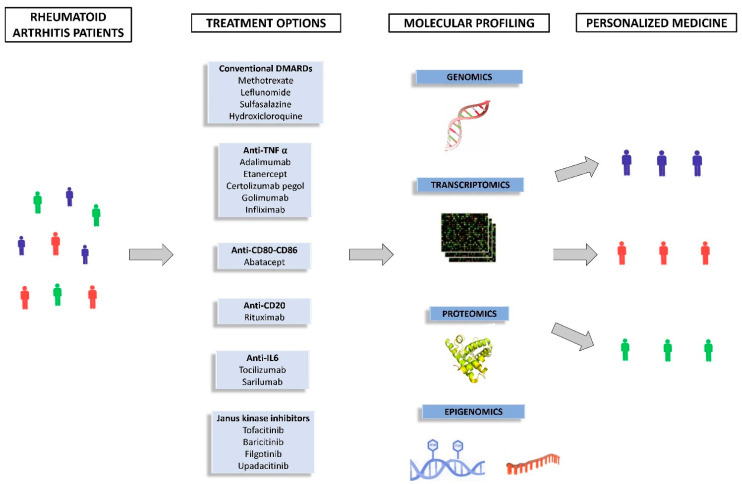
-Omic approaches for a personalized therapy.

Fortunately, today there are therapeutic alternatives available to reduce this excessive immune activation. According to the 2019 EULAR recommendations, different drugs that target relevant immune system molecules are currently used for the management of RA: non-steroidal anti-inflammatory drugs, glucocorticoids, immunosuppressants and disease-modifying antirheumatic drugs (DMARDs), such as methotrexate (MTX), leflunomide, sulfasalazine (SSZ) and hydroxycloroquine (HCQ), Janus kinase inhibitors (JAKi) (baricitinib, tofacitinib y upadacitinib) and biological DMARDs (TNF inhibitors (TNFi), abatacept (ABA), rituximab (RTX), tocilizumab (TCZ) and sarilumab) [4]. However, treatment response varies among patients and a significant percentage of them do not respond to first-line biological DMARDs, which results in high joint destruction and, consequently, a poor quality of life [5]. In this sense, recent evidence indicates that omics approaches are key to clarify the molecular mechanisms that influence treatment response (Figure 1) [6]. 

In recent years, several studies evaluating the role of -omic data in the response to RA drugs have been carried out. The aim of this review is to provide an updated overview of the potential role of omics approaches, specifically genomics, epigenomics, transcriptomics, and proteomics, to identify biomarkers to predict the clinical efficacy of therapies currently used in this disorder.

## 2. Methodology

### 2.1. Study Selection

The search was carried out in PubMed, Scopus and Google Scholar databases. The search strategy included studies published until 6 June 2022. The combinations of terms used were: “rheumatoid arthritis”, “response”, “anti-TNF”, “adalimumab”, “infliximab”, “golimumab”, “certolizumab”, “etanercept”, “abatacept “tocilizumab”, “sarilumab”, “rituximab”, “baricitinib”, “tofacitinib”, “filgotinib”, “upadacitinib”, “effectiveness”, “genomics”, “epigenomics” “methylation”, “proteomics”, “miRNA”, “proteomics”, “transcriptomics”, “single-cell”. 

This review included both observational cohort, case–control, cross-sectional studies, systematic reviews and meta-analyses (written in English). Animal and experimental model studies were excluded. In addition, studies in which treatment response results could not be extracted (responders/non-responders) were also excluded. Case reports, editorials, letters to the editor and conference proceedings were excluded from the review. Finally, a total of 72 references were included in this review.

### 2.2. Clinical Outcome 

The definitions of the different clinical outcomes that have been used in the studies evaluated are detailed below:

- Disease Activity Score 28-joint counts (DAS28) [7]: The result is calculated by using a special calculator that includes: Tender joint count (TJC) (of 28), swollen joint count (SJC) (of 28) and global health.
- EULAR (European Alliance of Associations for Rheumatology) response criteria [8]: This outcome classifies patients (good, moderate and non-responders) depending on the change in DAS28 and the level of disease activity reached during follow-up.
- CDAI (Clinical Disease Activity Index) [9]: This index is calculated using TJC (of 28), SJC (of 28), and patient and physician global assessment.
- SDAI (Simplified Disease Activity Index) [10]: Similar to the CDAI + C-Reactive Protein (CRP).
- HAQ-DIs (Health Assessment Questionnaire–Disability Index scores) [11]: a self-reported questionnaire covering 20 items in eight domains related to measuring difficulty in performing activities of daily living.
- ACR20 (American College of Rheumatology) [12]: The ACR20 is a composite measure defined as both improvement of 20% in the TJC and SWC, and improvement of 20% in three of the following five criteria: patient and physician global assessment, functional ability measure (HAQ), visual analog pain scale, and erythrocyte sedimentation rate or CRP.
- ACR/EULAR remission criteria [13]: This criteria include the index SDAI and CDAI and Boolean (SWJ (of 28), TJC (of 28), patient global assessment, and CRP).

A table including the abbreviations used throughout the manuscript has been added as Appendix A.

## 3. Pharmacogenomics Findings in Rheumatoid Arthritis

In recent years, genome-wide association studies (GWAS), which allow analyzing up to millions of single nucleotide polymorphisms (SNPs) through the whole genome, have emerged as an essential strategy to identify genetic risk loci involved in disease susceptibility. In addition, GWAS have also been applied to the identification of genetic polymorphisms involved in response to drugs. Specifically, 11 GWAS have evaluated the role of genetic variability in drug response for the different treatment used in RA.

To date, only two GWAS have analyzed the potential role of genetic variants in the response to MTX in RA patients. In this regard, a GWAS performed in 1424 RA patients from European ancestry identified an association that almost reached genome-wide significance (*p* = 9.8 × 10^−8^) between the *NRG3*-rs168201 polymorphism and the change in DAS28 at 6 months [14]. However, they could not replicate this association in two independent cohorts [14]. In addition, no genomic associations were found in a GWAS performed in an Indian cohort of 457 RA patients treated with MTX [15]. 

The largest replication study performed to date to identify genetic variants influencing response to MTX was performed in 915 patients. A total of 25 SNPs were evaluated (14 of them selected from candidate gene studies and 11 from GWAS). Patients carrying the A-allele of the *MTRR*-rs1801394 polymorphism showed a decrease in DAS28 after MTX treatment [16].

Recently, a systematic review that included 35 studies (34 candidate gene studies and the GWAS published by Senapati et al.) reported six promising SNPs after multiple correction. The genetic polymorphisms *ATIC*-rs7563206, *TYMS*-rs2847153 and *TYMS*-rs3786362 were associated with non-response to MTX. On the other hand, the genetic variants *SLC19A1*-rs1051266, *DHFR*-rs836788 and *TYMS*-rs2244500 were associated with response to this drug [17].

Genetic variability influencing response to TNFi has been more extensively studied during the last years. In 2010, a candidate gene study identified an association between the rs10919563 variant, located within *PTPRC*, and the response to TNFi [18]. Although subsequent GWASs have not detected this association, *PTPRC* currently represents the most replicated genetic biomarker of response to TNFi treatment in RA patients. In this regard, in 2016 a meta-analysis involving data from four studies (*n* = 2158) [18,19,20,21] showed that RA patients carrying the minor allele (A) of the *PTPRC* SNP presented a lower response to TNFi compared with those patients carrying the *PTPRC* major allele [22].

Two of the first GWASs performed in RA patients to evaluate the response to TNFi drugs (ADA, ETN, IFX) failed to identify significant differences between responder and non-responder patients, likely due to the low sample size included in these studies (*n* = 89 and *n* = 196) [23,24]. Although a genetic variant (rs3794271) located at the *PDE3A-SLCO1C1* locus did not reach genome-wide association with response to TNFi in the GWAS performed by Krintel et al. [24], this polymorphism reached statistical significance in a later study carried out in a Spanish cohort, when data were combined in a meta-analysis [25]. However, three subsequent replication studies, some of them performed in large cohorts of RA patients, failed to validate these results [26,27,28].

In a GWAS carried out by Plant et al. [29], four SNPs (rs12081765 and rs7305646, located in intergenic regions, and rs1532269 and rs17301249, located at *PDZ2D* and *EYA4*, respectively) were found putatively associated with good response to TNFi therapy at 6 months. Although they not reached genome-wide significance, associations were validated in two independent replication cohorts. In addition, they found that some of the SNPs associated in the discovery cohort showed opposite allelic effects in the replication cohort. This heterogeneity may be one of the reasons why the results obtained are not consistent across the different studies. A subsequent replication study performed in a cohort of 634 Spanish patients treated with ADA, ETN and IFX tried to validate these four associations [30]. After meta-analysis with previous GWAS data (*n* = 2.298) [24,29,31], none of these genetic variants was replicated. Only the SNP rs1532269, located at the *PDZD2* gene, yielded a suggestive association (*p* = 0.0033) with the response to TNFi.

Another GWAS was conducted in 882 RA patients treated with ADA, ETN and IFX through the Dutch Rheumatoid Arthritis Monitoring (DREAM) registry and the database of Apotheekzorg. They also included a replication cohort of 1821 patients from four cohorts. Again, none of the analyzed polymorphisms reached the genome-wide level of significance [31]. Moreover, they could not replicate the results obtained by previous GWASs [23,24,29]. 

In 2013, a large GWAS analyzed the response to therapy with various TNFi drugs (ADA, ETN and IFX) in 2706 RA patients collected through an international collaboration [32], including data of two previous GWASs [23,29]. Despite the higher statistical power of this study, no association with response to TNFi therapy was identified. However, when analyzing patients treated with each drug individually, a SNP located in the *CD84* gene, involved in the maturation and activation of T lymphocytes, was associated with the efficacy of ETN treatment. Furthermore, this polymorphism influenced gene expression levels, and a higher expression was associated with a better response to the drug. 

Finally, other GWAS performed in European population, including a discovery cohort of 375 RA patients and a replication cohort of 245 RA patients, identified the genetic polymorphism *MED15*-rs113878252 as a potential biomarker of response to ETN [33]. So far, only one GWAS trying to identify genetic biomarkers of response to ADA, ETN and IFX has been performed in patients from Asian origin, specifically from Japan. The genetic variant rs284511, which is close to the *MAP3K7* locus, was significantly associated with ΔDAS at 6 months in this population [34]. 

In recent years, several studies focused on validating genetic associations with treatment response have been published. A study of 566 RA patients of Spanish and Greek ancestries treated with IFX, ADA, and ETN analyzed 18 SNPs previously associated with TNFi response by GWAS and candidate gene studies, but they failed to replicate these associations [27]. A replication study conducted in 755 RA patients did not show an association between 12 GWAS-drawn SNPs [29,31,32] and the response to IFX, ADA and ETN [35]. However, stratification of patients according to each specific TNFi drug allowed the researchers to identify an association between the rs2378945 variant, located at the *NUBPL* locus, and a poor response to ETN [35]. In addition, a recent replication study of 28 GWAS-identified variants was performed in a cohort of 1361 RA patients treated with different TNFi from the REPAIR consortium and the DANBIO registry [28]. They also tried to validate the most interesting results by performing a meta-analysis with a cohort of 706 RA patients. An association between the rs7767069 SNP at the *LINC02549* locus and a lower response was found. Interestingly, the T-allele of this SNP correlated with a significantly increased number of T cells (CD45RO+CD45RA+), whereas patients carrying the TT genotype showed significantly increased serum levels of CD5 and CD6, which modulate T cells and certain subsets of B cells. Moreover, they also found that patients with positive rheumatoid factor carrying the G-allele of the *LRRC55* rs717117 polymorphism presented a lower response to TNFi treatment [28].

So far, only one GWAS has been performed evaluating the role of genetic variants influencing the response to CZP. A cohort of 302 RA patients from the REALISTIC trial was included in the study, and the response was evaluated at 6 and 12 months. No statistically significant differences were found [36], which may be due to the limited sample size of this study.

Finally, the role of genetics in TCZ response has also been evaluated. Specifically, a GWAS analyzing over 1600 RA patients treated with this drug identified 8 loci associated with the clinical efficacy of TCZ [37]. Notably, a subsequent replication study of this GWAS conducted in 79 RA patients treated with TCZ replicated two of these associations. In this sense, RA patients carrying the *GALNT18*-rs4910008 C-allele or the *CD69*-rs11052877 A-allele presented better clinical outcome [38].

Most of the GWAS carried out to date have been focused on genetic variants influencing the response to IFX, ADA and ETN. Despite their increasing statistical power and the subsequent replication studies performed in different cohorts, there is still no clear evidence of any biomarker that could predict the response to these drugs when evaluated together. However, more interesting results were found when GWAS data (Table 1) were stratified according to the type of drug, which indicates that within the same type of therapy, the genetic basis of the response to treatment may vary depending on the specific drug, and therefore, consideration of TNFi therapy globally may be masking association signals.

## 4. Epigenomics and Treatment Response

Epigenetics is the set of inherited alterations in gene expression that are stable and do not produce any abnormality in the nucleotide sequence of DNA [39]. These variations lead to chemical alterations in DNA molecules and histones that play a crucial role in regulating gene transcription. The main epigenetic mechanisms include DNA methylation, histone modifications and regulatory non-coding RNAs. To date, studies evaluating the role of epigenetic mechanisms in the response to drugs in RA has been mainly focused on DNA methylation and microRNAs.

### 4.1. DNA Methylation

DNA methylation takes place in many regions of the genome and is considered one of the main mechanisms of gene expression regulation [40]. It consists of the addition of methyl groups to cytosines to form 5-methylcytosines by DNA-methyltransferases (DNMT) at four sites: repetitive sequences, “CpG islands shores”, CpG islands in the promoter region, and gene bodies throughout the genome. This methylation blocks the transcription of genes in the promoter region [41], and in general, elevated levels of 5-methylcytosine in the CpG-rich promoter region are primarily related to transcriptional repression.

Most of the studies evaluating the role of global DNA methylation in RA treatment efficacy have been focused on MTX (Table 2). In this sense, several studies have explored the impact of MTX treatment in the methylation status in different cell types, finding conflicting results. After one month of MTX treatment, significant increases in 5 mC percentage were evident in three different cell types (T and B cells and monocytes) from 19 early RA patients, most of them showing a decreased RA activity; however, no differences were observed in natural killers (NK) or polymorphonuclear leukocytes [42]. These results could not be confirmed in a cohort of 16 RA patients after 3 months of MTX, sarilumab and/or JAKi treatment. In this case, an increased DNA methylation pattern was found in lymphocytes (*p* = 0.033) but not in specific blood cell types (CD4+, CD8+, CD19+ and CD14+) [43]. When evaluating methylation levels of two different CD4+ T cells subsets obtained from 11 RA patients in remission after MTX treatment, 80% of the differentially methylated positions (DMPs) detected in CD4+ memory T cells showed decreased methylation levels, whereas in CD4 naïve T cells, similar percentages were found for hypo- and hypermethylated positions. Two genes, *GRID2IP* and *PLEKHM1P1*, showed decreased methylation levels in CD4+ memory T cells and in CD4+ memory T cells and naïve CD4+ T cells, respectively [44]. In addition, no statistically significant differences in the methylation status were found in whole blood leukocytes [45] and PBMCs [46] from RA patients naïve to treatment before and after 3 months of MTX treatment.

Differences in the methylome of responder and non-responder RA patients have also been evaluated in several studies. In this regard, an increased global DNA methylation level was associated with a lack of MTX response after 3 months of treatment in 181 patients with early RA naive to therapy [45]. These same authors carried out a subsequent study to identify differentially methylated regions (DMRs), in addition to DMPs, in 69 PBMC samples from RA patients before and after 3 months of treatment with MTX and corticosteroids as monotherapy or in combination with other DMARDs (SSZ and HCQ). They identified 1309 DMRs, but none of them reached genome-wide significance [46]. An earlier study analyzing the baseline DNA methylation profile of T lymphocytes from 46 patients treated with MTX in combination with other DMARDs (SSZ and HCQ) revealed two CpG sites, located near the *ADAMTSL2* (hypermethylation) and *BTN3A2* (hypomethylation) genes, that, when evaluated in combination, were strongly associated with response to treatment at 6 months (area under the ROC Curve (AUC): 0.85) [49]. Interestingly, both molecules play an important role in the immune system regulation [51,52]. However, the study performed by Gosselt et al. failed to validate the association between the DNA methylation level of cg14345882 (*BTN3A2*) and treatment response [46].

Additionally, it has also been explored whether the methylation patterns in the first weeks of MTX treatment could predict the response to this drug in 68 RA patients. At 4 weeks, 2 CpG sites (cg21040096, nearest gene *RPH3AL* and cg09894276, nearest gene *WDR27*) were associated with treatment response at 6 months [50]. The modifications in the methylation patterns identified at 4 weeks could be used as a tool to prevent a possible early failure of MTX treatment allowing drug change or escalation, if necessary.

A few studies assessing the role of methylation in clinical response to other RA therapies have also been published. Analysis of baseline methylation patterns of responder (*n* = 36) and non-responder patients to etanercept (ETN) (*n* = 36) after 3 months of treatment allowed researchers to identify five hypermethylated positions in responders. Two of the five top CpGs (cg04857395, *p* = 1.39 × 10^−8^ and cg26401028, *p* = 1.69 × 10^−8^) were located within exon 7 of the *LRPAP1* gene that encodes a chaperone related to the transforming growth factor β activity [48], suggesting that this gene could be considered as a promising biomarker of ETN response. On the other hand, analysis of 26 patients treated with ETN or adalimumab (ADA) and 39 patients without biological agents failed to identify differences in the methylation profile and expression levels of *DNMT1* and methyl-CpG-binding domain 2 (*MBD2*) between patients treated with and without TNFi [47]. 

Changes in the global methylation profile before and after treatment as well as in the methylation level of specific genes have been proposed as markers of response or early failure to treatment with contradictory results. Well-powered studies analyzing patients with a combined treatment of biologics and MTX are necessary to validate whether these potential response biomarkers can be extrapolated to the clinical practice.

### 4.2. miRNA Profiling

MicroRNAs (miRNAs) are non-coding RNAs of approximately 21 nucleotides in length. These molecules function as posttranscriptional repressors of gene expression required for appropriate cellular processes [53]. Recent findings have suggested that the epigenetic dysregulation, in particular, changes in the miRNA expression profile, could influence on the immune dysregulation observed in RA patients [54]. 

Different studies have evaluated the miRNA profile of rheumatoid arthritis patients treated with different drugs, identifying several miRNAs with a potential role as predictors of treatment response. The main characteristics of the studies detailed below are summarized in Table 3.

In a cohort of 95 RA patients, serum levels of miR-146a-5p, miR-125b, miR-126-3p, miR-23-3p, miR-16-5p and miR-223-3p were upregulated in patients responding to a combination of TNFi (ADA, ETN and IFX)/DMARDs at 6 months according to EULAR criteria. In addition, ROC analysis showed that increased serum levels of miR-23-3p and miR-223-3p before starting therapy were indicative of non-response with high specificity when considered together (91.5%), suggesting that they could be used as predictors of response to TNFi/DMARDs therapy [55]. Additionally, a microarray assay performed in a cohort of 108 RA patients identified a total of 59 upregulated and 78 downregulated miRNAs in PBMCs from ETN responder patients after 24 weeks of treatment. Increased levels of miR-146a-5p and decreased levels of let-7a-5p were validated by qPCR in a cohort of 92 RA patients [57]. Apart from these miRNAs, clinical outcomes, such as C-reactive protein and biologic history, were independently associated with lower clinical response. Taken together, these four biomarkers showed a high ability to predict clinical response (AUC = 0.863) [57].

Both studies found a role of miR-146a-5p in the clinical efficacy of TNFi. This miRNA, through the NF-κB pathway, is able to stimulate the release of pro-inflammatory cytokines involved in RA pathogenesis, such as TNF-α, interleukin (IL)-1b, IL-17 [64] and IL-6 [65]. In addition, an increase in miR-146a-5p serum expression after 3 months of TNFi treatment was also observed in a cohort of 13 RA patients (*p* = 0.033) [56]. Moreover, miR-125b was also found to be upregulated in responder patients by miRNA profiling. Similar findings have been reported in additional studies. A high baseline serum expression level of miR-125b in 32 RA patients was associated with better response to RTX after 3 months (*p* = 0.002) [58]. Moreover, higher miR-125b and miR-125a basal expressions were associated with better outcome in 96 active RA patients after 24 weeks of infliximab (IFX) treatment [59]. Interestingly, miR-125b plays an important role in regulating different signaling pathways involved in RA development, such as inflammation by activation of NF-κB pathway [66].

In order to identify predictor biomarkers of response to ADA + MTX, an analysis of 91 specific miRNAs was performed in 89 RA patients from the OPERA study before and after 3 and 12 months of treatment initiation. A higher pre-treatment plasma level of miR-27a-3p was significantly associated with remission at 12 months whereas increased levels were found in non-responders at 3 months post treatment. After performing two multivariate miRNA models in pre-treatment samples based on 1 (miR-19b-3p) and 10 (miR-146b-5p, -19b-3p, -27a-3p, -16-5p, -423-5p, -27b-3p, -23a-3p, -106a-5p, -29b-3p, and -17-5p) miRNAs, they found ROC curves with AUC of 67% and 84%, respectively [61]. 

In addition, studies analyzing expression levels of individual miRNAs have also described interesting findings regarding treatment response. The combination of low expression of miR-22 and high expression of miR-886-3p was associated with EULAR good response in 180 treatment-naïve RA patients treated with ADA [60]. miR-29b, which has a role in resistance to apoptosis, showed a decreased expression in RA patients with effective IFX therapy, but not in those treated with TCZ, which suggested that miR-29b levels may be informative with regard to immunotherapy choice [67]. In addition, serum expression levels of miRNA-5196 were significantly increased in 10 RA patients after TNFi treatment, including golimumab (GOL), ADA, and CZP. Interestingly, changes in miRNA-5196 expression positively correlated with DAS28 score. Taking this into account, miRNA-5196 could serve as a predictive biomarker of response to these drugs [62]. On the other hand, the only study assessing the predictive value of miRNAs in response to JAKi did not identify differences between RA patients in remission and not in remission after treatment, which may be due to the reduced sample size of this study (*n* = 16) [63]. 

Although there is already consensus on the influence of some miRNAs, such as miR-125b and miR-146a-5p, on the response to the different drugs used in RA, validation studies are needed to be able to use them as response biomarkers.

## 5. Transcriptomic Biomarkers

There are many studies published to date that attempt to associate transcriptomic changes with the response to the different drugs used in RA (Table 4).

Most of the studies exploring the potential role of transcriptomics in predicting clinical efficacy of RA treatments have been focused on TNFi. Prior to 2010, several genome-wide gene expression analyses assessing TNFi therapy outcome were performed, but the differentially expressed genes between responder and non-responder patients identified among studies showed low overlap. In order to validate previously reported gene expression signatures, a subsequent study linked eight previously published transcript sets [88,89,90,91,92] predicting TNFi response to the expression values of 42 RA patients treated with IFX and ADA. This approach allowed validating one of these eight predictive expression profiles. Specifically, the set of 20 genes reported by Lequerré et al. obtained the best results, with a sensitivity of 71% and a specificity of 61% for classifying RA patients [71]. 

In addition, expression levels of *CD11c*, an integrin involved in a variety of cell-matrix and cell–cell adhesion functions [93], in monocytes of RA patients treated with ADA was associated with future response [92]; however, no association with response to ADA (*p* = 0.33) or ETN (*p* = 0.13) was found in PBMCs from 75 patients [76]. 

Controversial results between the expression of *CD39*, which is primarily expressed on activated lymphoid cells, and the response to treatment have also been found in several studies. Two different studies described a lower expression of *CD39* in poor responders to ADA (*n* = 70) [84] and MTX (*n* = 122) [94]. However, in a well-powered study including 2938 RA patients treated with all available TNFi drugs, a higher expression of *CD39* was associated with a worse response [81]. In the first case, authors evaluated the response using the EULAR criteria, while in the last study, the clinical response was evaluated using the swollen joint count outcome. In addition to the great difference in sample size between studies, the groups of patients included were also different due to reasons of clinical practice (±MTX), which make it difficult to discern which drug is responsible for the effect.

Although no baseline differences between responders and non-responders were found in 240 RA patients treated with different TNFi, when clinical efficacy was evaluated after 14 weeks of treatment, the good responders’ group showed higher expression levels in gene co-expression modules (GCM) related to plasma, B and T cells, major histocompatibility complex, ribosomal proteins and undetermined modules and downregulation of myeloid lineage, platelets and inflammation GCM [77]. A recent study also tried to identify changes in GCMs during early TNFi treatment in two cohorts of RA patients treated with MTX (85) and ADA (70). One module was associated with ADA response and presented IFN type 1 signaling pathway genes (*NFKBIE*, *IRF2BP2*, *MAPKAP-K2*, *IL1B* and *IFRD1*) [87]. Moreover, correlation between GCM and response to other drugs, such as TCZ ± MTX, was studied in 60 RA patients. Network analysis within CD4+ T cells identified two GCM in the TCZ+MTX arm, four in the TCZ arm and four in the MTX arm significantly associated with sustained drug-free remission (sDFR). These modules included relevant pathways such as nuclear-transcribed mRNA catabolic processes and ribosome (TCZ + MTX arm), granulocyte migration (TCZ arm) and response to bacterium, p53 and JAK-STAT signaling (MTX arm) [80]. No differences were found in CD14+ cells. 

By interrogating a synovial gene-expression dataset (GSE21537) including 62 patients treated with IFX, Dennis et al. found that good responder patients at week 16 presented higher baseline expression of a myeloid gene set (*p*= 0.011) and an enrichment in biological processes, such as classically activated M1 monocytes (*p*= 0.006) and angiogenesis (*p* = 0.018) [72]. Higher expression of *DERL-1*, a gene associated with autophagy, was associated with lack of IFX effectiveness in several cohorts of RA patients [86]. 

Expression of genes involved in the interferon (IFN) pathway has been widely studied in RA patients regardless of the administered drug. Patients who did not achieve IFX response showed an increased expression of the *OAS1* (*p* = 0.033) and *LGALS3BP* (*p* = 0.041) genes [69]. Another report of 51 RA patients evidenced that upregulation of three IFN response genes negatively predicted response to RTX at week 12 (9/24 versus 20/27 [R2 = 0.17; *p* = 0.01]) [68]. Moreover, at week 24, eight IFN type I response genes were associated with poor response to RTX in 14 RA patients (*p* = 0.0074) [70]. In addition, correlation between response to TNFi therapy and expression of IFN signaling pathway genes was studied in neutrophils from patients treated with ADA, ETN and GOL. Specifically, those patients classified in the IFN-high group expression showed a better response than patients in the IFN-low group (ΔDAS28, OR: 1.4; *p*= 0.05) [75]. An IFN pathway downregulation after 24 weeks of RTX treatment was also observed in 68 RA responders patients [73]. When an anti-IL6 treatment was considered (TCZ), a higher expression of four type I IFN genes (*IFI6*, *MT1G*, *MX2*, and *OASL*) was associated with good response [74]. In most of the studies, a high expression of genes involved in the IFN pathway was associated with a poor response to different RA treatments. Further studies with larger sample sizes and more homogeneous in terms of treatments will provide us a greater insight into how this pathway might predict response to RA treatments.

Different signaling pathways and biomarkers have been postulated as responsible for the response to ABA in patients with RA. In a study including 19 patients, responders to MTX/ABA (*n* = 14) by EULAR criteria showed a significant enrichment of six signaling pathways (T cell receptor signaling, proteasome, angiogenesis, apoptosis and two mRNA processes) (Table 4) [83]. Additionally, in 45 bDMARD-naïve RA patients, a decreased type I IFN score and higher expression levels of dendritic cell-related or type I IFN-related genes (*BATF2*, *LAMP3*, *CD83*, *CLEC4A*, *IDO1*, *IRF7*, *STAT1*, *STAT2 AND TNFSF10*) were identified after treatment in responder patients by EULAR criteria, suggesting that ABA produces a reduction in IFN type I activity [82]. Upregulated expression of genes related to elongation, arrest and recovery (OR: 6.85; *p* = 0.03309) as well as NK-cell-related genes (OR: 6.46; *p* = 0.00388) have been associated with the lack of ABA effectiveness by CDAI [78]. 

Recently, the analysis of synovial tissue of 50 RA patients evidenced that a higher baseline expression of genes involved in the myeloid leukocyte and T cell activation pathways was related to a better response after treatment with DMARDs (MTX, ADA, ABA, RTX, TCZ) [85], suggesting that a high baseline immune activation may predict the response to this therapy.

On the other hand, single-cell sequencing technology is a novel strategy that is being used to have a better understanding of the cell subpopulations involved in immune-mediated pathologies [95]. In the case of RA, several studies have characterized the role of different cell types in disease pathogenesis at the single cell level [96], which could lead to the identification of new surface molecules potentially useful as therapeutic targets. In this sense, the molecules involved in the development of the disease could be more selectively modulated. Unfortunately, to date, there is no single-cell study evaluating the response to treatment in patients with RA. It is to be expected that the application of this strategy to the identification of molecular profiles of response to drugs represents a great advance for personalized medicine.

A significant number of candidate genes, GCM and pathways have been proposed as biomarkers of treatment response in patients with RA (*TRAF6*, *CD11c*, *CD39*, *CHI3L1*, *EPPK1*, *CDC20*, *CXCR2*, *MPO*, *TNFAIP6*, *MYADM*, *FCGR2B*, *RFX2*, *IRF8* and *TAF1*, *FOXO4*, *TAF11*, IFN pathway and dendritic cell-related genes) but the differences in the characteristics of the studies published so far, in terms of type of tissue analyzed, time of response evaluation, sample size, therapy administered, patient characteristics, and analysis techniques, make it very difficult to reproduce the results obtained by the different studies.

## 6. Identification of Response Biomarkers by Proteomics

Evidence obtained in recent years suggests that proteome profiling also represents a useful tool for identifying response biomarkers. Table 5 summarized the more recent proteomics studies associated with the effectiveness to biological therapy in RA.

A serum proteome analysis was carried out in seven female RA patients treated with TCZ. Proteins expression levels were measured baseline and previous to the first and second dose of TCZ. A moderate/good response after two doses of TCZ was associated with an increased expression of 10 proteins, including apolipoproteins A-I, A-II, C-I, and C-II, Retinol binding protein 4, Selectin-L, Superoxide dismutase 3 or MCAM/MUC18/CD146). A decreased expression of seven proteins was observed after 2 months of TCZ treatment, such as pregnancy zone protein, a1-acid glycoprotein, C-reactive protein, haptoglobin, and the serine protease inhibitor clade A (a1-antitrypsin, leucine-rich a2-glycoprotein [97]. 

In addition to anti-citrullinated peptide antibodies (ACPA), RA patients can present other autoantibodies. In this sense, a study has analyzed the potential role of 376 different autoantibodies in treatment response by performing a multiplex bead-based assay in 286 RA patients treated with ADA or MTX [99]. By this approach, they identified two autoantibodies (citrullinated HNRNPA1 and citrullinated vimentin) significantly associated with treatment outcome by EULAR criteria at 3/6 months. In addition, the analysis of the ACPA patient subgroup identified an association of the presence of citrullinated CPSF6 with poor response at 3/6 months. Nevertheless, ACPA seropositivity is still the best marker to predict response to ADA/MTX.

Serum protein profiles at weeks 0 and 14 were investigated in 20 RA patients treated with IFX after one month of MTX and leflunomide. A total of 5 from the 13 differentially expressed proteins that overlapped (fibrinogen beta chain, haptoglobin, testicular tissue protein Li 70, C-reactive protein and serotransferrin) were validated, but only the serotransferrin protein was significant after verification by parallel reaction monitoring. Indeed, this protein involved in the hypoxia-inducible factor-1 pathway and ferroptosis, was upregulated in the responder patients (*n* = 15) and downregulated in the non-responder patients (*n* = 5) treated with IFX, MTX and leflunomide by EULAR criteria after 14 weeks of therapy, indicating the possible role of serotransferrin in resistance to these medications [100]. Although haptoglobin could not be validated as a response biomarker in this study, high levels of haptoglobin were associated with a good response in a cohort of 50 RA patients baseline and after 6 months of ETN treatment [98].

## 7. Multi-Omic Approaches for Response Prediction

Although individual -omic approaches have been useful in the identification of molecular biomarkers of response prediction, using integrative approaches that combine different -omic layers result essential to better understand how different mechanisms act in a complementary way to modify treatment response. Several studies that jointly evaluate the effect of the genome, methylome, transcriptome and proteome on the response to RA treatment have been published in recent years. The main characteristics of the studies detailed below are summarized in Table 6.

The effect on gene expression and protein levels of three drugs with different mechanisms of action has been analyzed in 45 patients with RA and 35 controls. At week 24, 600 transcripts were differentially expressed (FDR < 0.05) in patients treated with IFX or TCZ but not with MTX, and this effect was greater for TCZ than for IFX. In addition, in the case of TCZ, most changes in gene expression were in the direction toward the healthy state. In addition, when they studied the influence of TCZ and IFX on molecular remission at 14 weeks, TCZ and IFX, but not MTX, were able to achieve molecular remission at the protein level and, again, this effect was greater for TCZ. Although it would be necessary to carry out additional studies to confirm these results, it seems that TCZ has a more powerful effect than IFX and normalizes the molecular profiles of RA patients at transcriptome and protein levels. Interestingly, these drugs also produced transcriptional changes mainly in genes that were expressed at high or low levels in neutrophils, suggesting that the neutrophil signature was normalized by the drug treatments [102]. 

By integrating transcriptomic and genomic data, Aterido et al. identified a gene signature associated with TNFi response. The analysis of transcriptomic data from the synovium of 11 RA patients yielded an association between 13 GCMs and the response to TNFi at week 14. Notably, two of these GCMs were also associated with the response to ADA and IFX at the genetic level in GWAS data from a Spanish cohort (348 patients). In addition, the ADA-associated module, which was significantly enriched for genes involved in the nucleotide metabolism and epigenetic marks from CD4+ regulatory T cells, was validated in an independent GWAS set (2706 patients) (*p* < 0.05) [101], displaying the relevant role of these cells in mediating the response to TNFi [101].

Recently, a study evaluated gene expression and protein levels in PBMCs from 39 female RA patients and developed machine learning models to predict treatment response [104]. Analysis of baseline gene expression levels identified 192 differentially expressed genes between future responders and non-responders. Specifically, the genes *EPPK1*, *BCL6-AS1* and *CDC20* showed the highest differences between both subgroups. Some changes were also showed during treatment; in this regard, *CXCR2, MPO, TNFAIP6* and *MYADM* were downregulated by treatment and *FCGR2B* appeared to be upregulated. Furthermore, some proteins, as CRP, IL-6, MMP-1, SAA, TNF-RI, VEGF, YKL-40, MIP-1 beta and MIG, were significantly suppressed in responders (FDR < 0.05) during anti-TNF treatment. In addition, a suppression of gene expression levels of *CHI3L1* was evident upon TNFi treatment. Interestingly, this gene encodes YKL-40, one of the proteins that showed decreased levels after treatment [104]. Finally, they developed machine learning models that showed high prediction capacity in classifying non-responders RA patients before TNFi treatment, especially the model based on transcriptomic data (AUC = 0.81).

Differentially expressed genes and DMPs were found between responders and non-responders in PBMcs, monocytes and CD4+ T cells of 80 RA patients treated with ADA or ETN. In PBMCs, large differences were found in the gene expression profiles of responder and non-responder patients treated with ADA or ETN, including some genes involved in DNA nucleotide binding, specifically *RFX2*, *IRF8* and *TAF1* for the ADA cohort, and *FOXO4* and *TAF11* for the ETN cohort. *TRAF6* involved in TNF receptor signaling, was only differentially expressed between responders and non-responders in the ETN cohort [103]. Interestingly, a low percentage of these differentially expressed genes overlap between both treatments. DMPs were strongly hypermethylated in ETN responders (76%) compared to ADA responders (46%). In addition, genes associated with the TNF signaling pathway were dysregulated in CD4+ T cells but not in monocytes of patients treated with ADA, which may be due to a clearer molecular TNF signaling signature associated with CD4+ T cells. CD4+ T cells from ETN responders showed upregulated genes in the FoxO signaling pathway and downregulated genes in the NOD-like receptor and JAK/STAT signaling pathways. With these results, the authors performed different machine learning models trying to predict the response to these drugs. The final model based on the best expression and methylation models predicted that approximately 30% of patients will not respond to ADA or ETN [103]. 

The DNA methylation profile in PBMCs and six immune system cells was evaluated recently in a discovery cohort of 62 RA and a validation cohort of 60 patients treated with different anti-TNF. At baseline after validation, 11 CpG sites from monocytes, 3 from NK, 2 from CD4+ T cells and 2 from neutrophils were associated with response. At week 12, 38 CpGs replicated in NK cells, 24 in neutrophils, 21 in B cells, 19 in monocytes, 13 in CD8+ T cells and 6 in CD4+ T cells were also associated with response. The cell-type deconvolution approach identified CpG sites in CD4+ T cells, NK cells, neutrophils and monocytes that were significantly associated with the response to TNFi. They found that from the 99 pathways modified at the epigenetic level in responders, 73 (73.7%) were also significantly altered at the transcriptomic level compared to 11 pathways statistically modified at the transcriptomic level from the 57 differentially methylated pathways in non-responders [105].

## 8. Conclusions

In contrast to studies focused on analyzing specific candidate molecules, -omic strategies allow the evaluation of a large number of potential biomarkers, thus representing a powerful tool to identify molecular signatures predicting treatment response. In this regard, the application of genomic, epigenomic, transcriptomic, and proteomic approaches to the discovery of response biomarkers has yielded relevant findings in RA.

However, despite the great effort carried out in recent years to clarify the role of epigenetic modifications, genes and proteins in the ineffectiveness of RA treatments, to date, there are still no validated biomarkers of response to the drugs currently used in this pathology. In this sense, the inconsistent results observed appear to be largely due to the heterogeneity across studies with respect to different variables that directly influence treatment outcome, such as patient phenotype, timing of sample collection, type of outcome, or response criteria. Therefore, it results essential to perform well-powered validation studies with homogenized conditions in order to be able to identify reliable treatment response biomarkers. In addition, -omic studies have evidenced significant differences in the molecular profiles associated with treatment response for the different drugs used in RA, as in the case of anti-TNF drugs, and also for different cell types. This highlights the importance of analyzing each drug independently as well as in individual cell types.

Therefore, global, single-cell and cell type-specific studies analyzing response to the complete therapeutical arsenal used in RA, including both biological and targeted synthetic therapies (sarilumab and JAK inhibitors), are greatly needed.

In addition, due to the complexity of the molecular mechanisms involved in treatment response, studies integrating different omics layers are essential to unravel the molecular network that determines clinical response, as well as to develop predictive models that allow the identification of RA patients with a greater probability of responding efficiently to treatment, thus leading to better clinical management of these patients.

## Figures and Tables

**Table 1:** GWAS and replication studies and response to DMARDs in rheumatoid arthritis patients.

Drug	Clinical Outcome	Discovery Cohort (*n*)	Replication Cohort (*n*)	Main Associations	*p*-Value	Replication	Reference
MTX	∆DAS28 at 6 months	1424	429 177	*NRG3 (rs168201)*	**9.8 × 10^−8^**	-	[14]
MTX	∆DAS28 at 6 months	457	-	*ARL14|PPM1L (rs7624766)*	3.9 × 10^−7^	-	[15]
ADA, ETN, IFX	∆DAS28/EULAR at 3 months	89	-	*MAFB* (rs6028945)	2 × 10^−7^	No [28,33]	[23]
				*IFNK* (rs7046653)	5 × 10^−7^	-	
ADA, ETN, IFX	∆DAS28/EULAR at 3 months	196	-	*NR2F2* (rs10520789)	6 × 10^−7^	No [28]	[24]
				*PDE3A-SLCO1C1* (rs3794271)	3.5 × 10^−6^	Yes [25] No [26,27,28]	
ADA, ETN, IFX	∆DAS28 at 6 months	566	774	*EYA* (rs17301249)	6 × 10^−5^	No [30]	[29]
				Intergenic region (rs12081765)	7 × 10^−4^	No [30]	
				*PDZD2* (rs1532269)	7 × 10^−4^	Yes [30]	
				Intergenic region (rs7305646)	1 × 10^−4^	No [30]	
ADA, ETN, IFX	∆DAS28 at 3 months	882	1821	Intergenic region (rs4411591)	5 × 10^−5^	Yes [35]	[31]
ADA, ETN, IFX	∆DAS28/EULAR from 3 to 12 months	2706	290	*CD84* (rs6427528)	**8 × 10^−8^**	Yes [35]	[32]
ADA, ETN, IFX	∆DAS28 at 12 weeks	375	245	*MED15* (rs113878252)	**1.2 × 10^−8^**	-	[33]
ADA, ETN, IFX	∆DAS28 at 6 months	487	-	rs284511	**2.5 × 10^−8^**	No [27]	[34]
CZP	ΔACR20, ΔDAS28 at week 6 and ΔDAS28 at week 12	302	-	rs12287315	**5.7 × 10^−8^**	-	[36]
				rs35355083	1.5 × 10^−7^		
TCZ	∆DAS28 at 4 months ACR20 at 6 months	1157	526	*CD69* (rs11052877)	4 × 10^−3^	Yes [38]	[37]

ACR (American College of Rheumatology); ADA (Adalimumab); CZP (Certolizumab pegol); DAS (Disease Activity Score); ETN (Etanercept); IFX (Infliximab); MTX (Methotrexate); TCZ (Tocilizumab).

**Table 2:** Methylation studies and response to DMARDs in rheumatoid arthritis patients.

Study	Patients (*n*)	Drug	Sample	Time Sample	Outcome
Liu et al., 2011 [47]	65	ETN or ADA (26)	Peripheral blood	Baseline	DAS28
De Andrés et al., 2015 [42]	19 early RA patients	MTX (12 GR, 1 MR, 2 NR) /17 controls	T, B, NK, monocytes and polymorphonuclear leukocytes from whole blood	Baseline/ after 1 month	DAS28 at 6 months
Plant et al., 2016 [48]	72	ETN (36 GR/36 PR)	Whole blood	Baseline	DAS28 at 3 months
Glossop et al., 2017 [49]	46	MTX, SSZ and HCQ (35 GR/11 NR)	Whole blood	Baseline	EULAR criteria at 6 months
Gosselt et al., 2019 [45]	181	MTX or MTX + SSZ + HCQ + corticosteroids (140 MR/GR and 41 NR)	Whole blood leukocytes	Baseline and at 3 months	DAS28
Liebold et al., 2021 [43]	16 RA 17 controls	MTX, sarilumab, Janus kinase inhibitors (8 GR-MR/8 NR)	Peripheral blood and CD4+, CD8+, CD14+ and CD19+	Baseline/3 months	DAS28-ESR DAS28-CRP
Guderud et al., 2020 [44]	72	MTX (36 GR + 36 PR)	Whole blood	Baseline and 4 weeks after MTX	EULAR criteria at 6 months
Nair et al., 2020 [50]	68	MTX (34 GR + 34 PR)	Whole blood	Baseline and 4 weeks after MTX	DAS28 at 6 months
Gosselt et al., 2021 [46]	69	MTX or MTX + SSZ + HCQ + corticosteroids	Whole blood	Baseline	DAS28 at 3 months

ADA (Adalimumab); CRP (C-Reactive Protein); DAS28 (Disease Activity Score in 28 joints); ETN (Etanercept); ESR (Erythrocyte Sedimentation Rate); GR (Good Response); HCQ (Hydroxicloroquine); MTX (Methotrexate); MR (Moderate Response); NR (Non-Responders); PR (Poor Response); SJC (Swollen Joint Count); SSZ (Sulphasalazine); TJC (Tender Joint Count).

**Table 3:** MiRNA studies and response to DMARDs in rheumatoid arthritis patients.

Study	Patients (*n*)	Drug (*n* Patients)	Response (*n*)	Sample	Time Sample	Outcome
Castro Villegas et al., 2015 [55]	Study cohort (10); Replication cohort (85)	ADA (15), ETN (25) and IFX (55)	GR (85) NR (10)	Serum	Baseline and at 6 months	EULAR criteria at 6 months
Bogunia-Kubik et al., 2016 [56]	13	anti-TNF-α	Not specified	Serum	Before and after 3 months of TNFi	EULAR criteria 3 months
Liu et al., 2019 [57]	Study cohort (16); Replication cohort (92)	ETN	8 GR; 8 NR 60 GR; 32 NR	PBMCs	Baseline	EULAR criteria at week 24
Duroux-Richard et al., 2014 [58]	32	RTX	16 GR; 16 NR	Blood (16) and serum samples (32)	Baseline	EULAR criteria at 3 months
Cheng et al., 2020 [59]	96	IFX	69 GR; 27 NR	Peripheral blood samples	Baseline, 4, 12 and 24 weeks	EULAR criteria at week 24
Krintel et al., 2015 [60]	180	ADA (89) or ADA ± MTX (91)				EULAR criteria
Sode et al., 2018 [61]	89	ADA + MTX (89)	ADA + MTX: 40 GR; 46 NR	Plasma	Baseline and at 3 months	ACR/EULAR remission at 3 and 12 months
Ciechomska et al., 2018 [62]	10	ETN (7) ADA (3)	Not specified	Serum	Baseline and after TNFi	DAS28
Fernandez-Ruiz et al., 2018 [63]	16	Tofacitinib	10 Remission; 6 No remission	Blood	At the first month after the last dose of tofacitinib	Remission ((DAS28) <2.6 and no swollen joints)

ACR (American College of Rheumatology); ADA (Adalimumab); DAS (Disease Activity Score); ETN (Etanercept); GR (Good Response); IFX (Infliximab); MTX (Methotrexate); NR (Non-Responders); PBMC (Peripheral Blood Mononuclear Cell); RTX (Rituximab).

**Table 4:** Studies that Explore Transcriptomic Biomarkers and Response to DMARDs in RA Patients.

Study	Patients (*n*)	Drug (*n* Patients)	Response	Sample	Time Sample	Outcome
Thurlings et al., 2010 [68]	51	RTX	Not specified	PBMC	Baseline	EULAR criteria at weeks 12 and 24
Van Baarsen et al., 2010 [69]	33	IFX	(12 GR and 6 PR)	Whole blood	Before/after 1 month	DAS, tender joint counts and HAQ-DIs criteria at week 16
Raterman et al., 2012 [70]	14	RTX	8 GR; 6 NR	Whole blood	Baseline	EULAR at week 24
Toonen et al., 2012 [71]	42	IFX (27) or ADA (15)	(18 GR and 24 NR)	Whole blood	Baseline	EULAR criteria at week 14
Glynn Dennis et al., 2014 [72]	GSE21537 dataset (62)	IFX	Not specified	Synovial	Baseline	EULAR at week 16
Sellam et al., 2014 [73]	68	RTX	44 GR; 24 NR	PBMCs	Baseline and 24 weeks	EULAR at week 24
Sanayama et al., 2014 [74]	40 + 20	TCZ	GR 29 NR 8 GR 15 NR 5	PBMC	Baseline, 3 and 6 months	physician’s global assessment and CDAI at 6 months
Wright et al., 2015 [75]	20	ADA (13), ETN (5), GOL (2)	5 GR; 13 MR; 2 NR	Neutrophils	Baseline	DAS28 at week 12
Smith et al., 2015 [76]	75	ADA (25) ETN (50)	ADA (16 GR, 9 NR) ETN (25 GR, 25 NR)	Whole blood	Baseline	EULAR criteria at month 3
Oswald et al., 2015 [77]	240	ABCoN (IFX 20, ETN 21, ADA 9) GO-FURTHER (GOL 72) BATTER-UP (IFX 23, ETN 31, GOL 9, ADA 41, CZP 14)	ABConN (GR 35, NR 15) GO-FURTHER (GR 66, NR 6) BATTER-UP (GR 79, NR 39)	Whole blood	Baseline/after 14 weeks	EULAR at 14 weeks
Nakamura et al., 2016 [78]	209	IFX (140), TCZ (38), or ABA (31)	IFX (30% REM), TCZ (21.1% REM), ABA (22.6% REM)	Whole blood	Baseline	CDAI at 6 months
Wampler Muskardin et al., 2016 [79]	Test cohort:32 (ABCoN) Validation cohort: 92 (TETRAD registry)	IFX (19), ADA (37), ETN (60), GOL (2), CZP (6)	Test cohort: 13 NR and 19 GR Validation cohort: 44 NR, 30 MR and 18 GR	Serum sample	Baseline	EULAR at 14 weeks EULAR at 12 weeks
Teitsma et al., 2017 [80]	60	MTX + TCZ (19) MTX + TCZ (24) MTX + TCZ (17)	14 sDFR 5 control 13 sDFR 11 controls 10 sDFR 7 controls	Whole blood	Baseline	sDFR
Sipiliopoulou et al., 2019 [81]	2938 (BRAGGSS, DREAM, EIRA, ReAct, WTCCC, Other cohorts)	IFX (792), ADA (1255), ETN (721), GOL (17), CZP (34)	Not specified	Whole blood	Baseline	ESR and SJC baseline and between 3–6 months after treatment
Yokoyama-Kokuryo et al., 2020 [82]	45	ABA ± MTX	27 GR; 8 MR/NR	Whole blood	Baseline and 6 months	EULAR at 6 months
Derambure et al., 2020 [83]	19	ABA + MTX	14 GR; 5 NR	Whole blood	Baseline and 6 months	DAS28-CRP at 6 months
Oliver et al., 2021 [84]	70	ADA	50 GR; 20 NR	Whole blood	Baseline and 3 months	EULAR at 3 months
Triaille et al., 2021 [85]	50	MTX, ADA, ABA, RTX, TCZ	Not specified	Synovial tissue	Baseline and after 16 weeks	EULAR at 16 weeks
Cai et al., 2022 [86]	Test cohorts: GSE58795, GSE78068 Validation cohorts: GSE77298, GSE55457, and GSE89408 datasets	IFX	GSE58795 36 GR; 23 NR GSE78068 42 GR; 98 NR GSE77298: 16 RA GSE55457: 13 RA GSE89408: 152 RA	Whole blood Synovium	Baseline	ESR and CRP
Sutcliffe et al., 2022 [87]	155 RAMS (MTX) BRAGGSS cohort (ADA)	MTX (85) or ADA (70)	42 GR; 43 NR 50 GR; 20 NR	Whole blood	Baseline and at 4 weeks Baseline and at 3 months	EULAR criteria after 3 months EULAR criteria after 6 months

ABA (Abatacept); ADA (Adalimumab); CDAI (Clinical Disease Activity Index); CRP (C-Reactive Protein); CZP (Certolizumab pegol); DAS (Disease Activity Score); ESR (Erythrocyte Sedimentation Rate); ETN (Etanercept); GOL (Golimumab); GR (Good Response); HAQ-DIs (Health Assessment Questionnaire–Disability Index scores); IFX (Infliximab); MTX (Methotrexate); NR (Non-Responders); MR (Moderate Response); REM (Remission); RTX (Rituximab); NON-REM (Non-Remission); sDFR (sustained Drug-Free Remission); SJC (Swollen Joint Count); TCZ (Tocilizumab).

**Table 5:** Summary of the proteomics studies associated with the effectiveness to biological therapy in RA.

Study	Patients (*n*)	Drug (*n* Patients)	Response	Sample	Time Sample	Outcome
Yanagida et al., 2013 [97]	7	TCZ	7 MR or GR	Serum	Baseline, 4 and 8 weeks	DAS28 (Baseline, 4 and 8 weeks)
Blaschke et al., 2015 [98]	50	ETN	31 GR, 19 NR	Serum	Baseline/after 12 and 24 weeks	EULAR criteria at 6 months
Ling et al., 2020 [99]	286	BRAGGSS cohort- ADA (150) RAMS cohort- MTX (136)	ADA: 58 GR, 58 MR and 34 PR. MTX: 59 GR, 2 MR and 75 PR	Serum	Baseline	EULAR criteria at 3 months (BRAGGSS) EULAR criteria at 6 months (RAMS)
Chen et al., 2021 [100]	20	IFX + MTX + Leflunomide	5 NR, 15 GR	Serum	Baseline and after 14 weeks	EULAR criteria after 14 weeks

ADA (Adalimumab); DAS (Disease Activity Score); ETN (Etanercept); GR (Good Responders); IFX (Infliximab); MTX (methotrexate); MR (Moderate Responders); NR (Non-Responders); TCZ (Tocilizumab).

**Table 6:** Multi-omic studies and response to DMARDs in rheumatoid arthritis patients.

Study	Patients (*n*)	Drug (*n* Patients)	Response	Sample	Time Sample	Outcome	Omics
Aterido et al., 2019 [101]	11	ADA	GR 5 NR 3 MR 3	Synovial biopsies	baseline	EULAR criteria at week 14	Transcriptomic and genomic
Tasaki et al., 2018 [102]	34 RA 35 controls	MTX (21), TCZ (13) and IFX (18)	IR: MTX (11), TCZ (3) and IFX (8) GR: MTX (10), TCZ (10) and IFX (10)	26 cell types from whole blood	Baseline, 4, 8, 12 and 24 weeks	EULAR criteria at week 24 (DAS28-ESR)	Transcriptomics and proteomics
Tao et al., 2021 [103]	80	ETN (38) or ADA (42)	ADA (20 GR/MR; 18 NR) ETN (19GR/MR; 23 NR)	CD14+, CD4+ from whole blood	Baseline	EULAR criteria at 6 months	Transcriptomics and epigenomics
Yoosuf et al., 2022 [104]	39 female	IFX (16), ADA (11), ETN (8), GOL (2), CZP (2)	23 (GR/MR); 16 (NR)	PBMCs from whole blood	Baseline and 3 months	EULAR criteria	Transcriptomics and proteomics
Julià et al., 2022 [105]	Discovery cohort (62) Validation cohort (60)	ADA (5), CZP (10), ETN (34), GOL (12), IFX (1) ADA (7), CZP (13), ETN (31), GOL (9), IFX (0)	Week 0: GR (50); NR (12); Week 12: GR (44); NR (7) Week 0: GR (49); NR (10); Week 12: GR (48); NR (10)	Whole blood and neutrophils, macrophages, CD4+ T, CD8+ T, B and NK cells	Baseline and at week 12	EULAR criteria at week 12	Transcriptomics and epigenomics

ADA (Adalimumab); CZP (Certolizumab pegol); DAS (Disease Activity Score); ESR (Erythrocyte Sedimentation Rate); ETN (Etanercept); GOL (Golimumab); GR (Good Response); IFX (Infliximab); IR (Inadequate Response); MR (Moderate Response) MTX (Methotrexate); NR (Non-Responders); PBMC (Peripheral Blood Mononuclear Cell); TCZ (tocilizumab).

## Data Availability

Data sharing is not applicable.

## Appendix A

**Table A1:** Abbreviations.

ABA (Abatacept)	JAKi (Janus kinase inhibitors)
ACPA (Anti-Citrullinated Peptide Antibodies)	IFN (Interferon)
ACR (American College of Rheumatology)	IFX (Infliximab)
ADA (Adalimumab)	MTX (Methotrexate)
CDAI (Clinical Disease Activity Index)	NK (Natural Killers)
CPZ (Certolizumab pegol)	RDM (Region Differentially Methylated)
CRP (C-Reactive Protein)	RA (Rheumatoid Arthritis)
DAS28 (Disease Activity Score 28-joint counts)	RTX (Rituximab)
DMARDs (Disease-Modifying Antirheumatic Drugs)	SDAI (Simplified Disease Activity Index)
DMRs (Differentially Methylated Regions)	TCZ (Tocilizumab)
DNMT (DNA-Methyltransferases)	TNFi (TNF inhibitors)
ETN (Etanercept)	Treg (Regulatory T cell)
EULAR (European Alliance of Associations for Rheumatology)	PBMC (Peripheral Blood Mononuclear Cell)
GCM (Gene Coexpression Modules)	PDM (Positions Differentially Methylated)
GWAS (Genome-Wide Association Study)	RTX (Rituximab)
GOL (Golimumab)	sDFR (Sustained Drug-Free Remission)
HAQ-DIs (Health Assessment Questionnaire–Disability Index scores)	SNP (Single Nucleotide Polymorphisms)
HCQ (Hydroxicloroquine)	SSZ (Sulfasalazine)
